# Cross-sectional analysis of expressive and receptive language skills in Smith-Lemli-Opitz syndrome (SLOS)

**DOI:** 10.1007/s44162-025-00119-5

**Published:** 2025-09-19

**Authors:** Stephanie M. Morris, Elaine Tierney

**Affiliations:** 1 Department of Neurology, Kennedy Krieger Institute and Johns Hopkins School of Medicine, Baltimore, MD USA; 2 Department of Psychiatry and Behavioral Sciences, Kennedy Krieger Institute and Johns Hopkins School of Medicine, Baltimore, MD USA

**Keywords:** Smith-Lemli-Opitz syndrome (SLOS), Speech development, Cholesterol metabolism, Autism spectrum disorder

## Abstract

**Purpose:**

This study investigates receptive and expressive language abilities in individuals with Smith-Lemli-Opitz syndrome (SLOS) and examines how these are associated with intellectual functioning, sex, autism spectrum disorder (ASD) diagnosis, and biochemical markers of cholesterol metabolism.

**Methods:**

Participants (ages 4–18) with mild to classic SLOS were enrolled from a double-blind, placebo-controlled simvastatin trial. Receptive and expressive language scores were assessed using the Peabody Picture Vocabulary Test, Third Edition (PPVT-3), the Expressive One-Word Picture Vocabulary Test, 2000 Edition (EOWPVT-2000), and the MacArthur Communicative Developmental Inventories (MCDI). Intellectual ability and adaptive functioning were measured using the Stanford-Binet Intelligence Scales, Fifth Edition (SB-5) and Vineland Adaptive Behavior Scales, Third Edition (VABS-3). The SLOS Severity Scale (SSS) quantified disease severity. Associations with plasma and CSF sterol biomarkers (cholesterol, 7-dehydrocholesterol [7-DHC], 8-dehydrocholesterol [8-DHC]) were examined using nonparametric statistics with correction for multiple comparisons.

**Results:**

Twenty-one participants (mean age 7.85 years) had complete data; 71.4% had a diagnosis of ASD. Receptive and expressive language scores correlated with IQ and adaptive functioning. Receptive vocabulary scores were significantly negatively associated with disease severity, plasma 7-DHC and 8-DHC, and CSF 7-DHC. Expressive vocabulary scores also declined with increasing disease severity, but associations with sterol biomarkers were not significant. ASD was linked to higher rates of non-scorable assessments, though did not fully explain floor effects. No sex differences were found.

**Conclusions:**

Language impairment in SLOS reflects contributions from disease severity, disrupted cholesterol metabolism, and ASD. Receptive language showed stronger biomarker associations, while expressive impairments were more pervasive. Integrating clinical, biochemical, and caregiver-report tools is critical for comprehensive assessment of individuals with SLOS.

## Introduction

Smith-Lemli-Opitz syndrome (SLOS) is a rare genetic disorder caused by variants in the *DHCR7* gene (OMIM:270400), which encodes 7-dehydrocholesterol reductase, the enzyme responsible for the final step of cholesterol biosynthesis. This enzymatic deficiency results in decreased cholesterol levels and accumulation of precursor sterols, particularly 7-dehydrocholesterol (7-DHC) and 8-dehydrocholesterol (8-DHC), in affected individuals (Irons, 8). Clinically, SLOS is characterized by a variable constellation of clinical features, including intellectual disability, congenital malformations, growth retardation, and distinct facial features. Communication impairments, especially in speech and language, are commonly reported [10] and contribute to the broader developmental challenges experienced by affected individuals. In addition, autism spectrum disorder (ASD) is highly prevalent in this population, affecting approximately 50% of individuals (Tierney, 18), further increasing the heterogeneity in cognitive and language outcomes.

Despite early recognition of language impairments in SLOS, few studies have systematically characterized the nature of expressive and receptive language deficits or examined their relationship to intellectual functioning, ASD diagnosis, disease severity, and biochemical markers of disrupted cholesterol metabolism. Emerging evidence suggests that language development in SLOS may be influenced not only by cognitive delays but also by structural brain abnormalities [12], auditory dysfunction [23], and direct effects of cholesterol deficiency on neuronal development and function [18]. However, the specific contributions of these biological and developmental factors to language outcomes remain poorly understood.

Although speech and language abilities can vary widely among individuals with SLOS [18], standardized language and cognitive assessments are often challenging to complete due to profound impairments, frequently resulting in floor effects or non-scorable data. These challenges limit the utility of conventional tests for accurately characterizing abilities across the full range of functioning. As a result, caregiver-reported measures may be especially valuable in capturing language abilities in individuals who cannot complete direct assessments. Moreover, adaptive functioning may serve as an important proxy for developmental capacity and test feasibility, although these associations have not been formally evaluated in this population.

Biochemical abnormalities are a core feature of SLOS, but the relationship between specific sterol markers and language outcomes remains underexplored. While prior work has focused on plasma cholesterol levels [17], the accumulation of precursor sterols, such as 7-DHC and 8-DHC, may more sensitively reflect the severity of metabolic disruption and its impact on neurodevelopment. Understanding how these markers correlate with language performance could enhance their utility as biomarkers of functional outcomes and informed tailored interventions.

In this regard, the present study aims to characterize receptive and expressive language performance in children and adolescents with SLOS and to examine associations with intellectual functioning (IQ), co-occurring ASD, disease severity, and biochemical markers of cholesterol metabolism, including plasma and CSF levels of cholesterol, 7-DHC, and 8-DHC. Additionally, this study evaluates the utility of adaptive functioning in predicting language test feasibility and performance. By integrating direct testing, caregiver-report measures, and biomarker data, this study seeks to advance our understanding of the mechanisms underlying language impairment in this rare neurodevelopmental disorder and to inform more inclusive, ecologically valid assessment strategies for use in clinical and research settings.

## Methods

Participants were recruited from a double-blind, placebo-controlled clinical trial evaluating simvastatin therapy in individuals with SLOS [21]. The cohort included 23 participants aged 4 to 18 years with clinically mild to classic SLOS. All participants completed standardized assessments of receptive and expressive language using the Peabody Picture Vocabulary Test, Third Edition (PPVT-3 [5]) and the Expressive One-Word Picture Vocabulary Test, 2000 Edition [3]. Standard scores (SS) below the test floor (i.e., < 55) were assigned a score of 54 for analysis purposes. PPVT and EOWPVT were considered non-scorable (NSB) when participants could not establish a basal level, preventing valid score generation.

The MacArthur Communicative Developmental Inventories (MCDI; [7]) were used as an alternative measure of expressive and receptive language in this cohort. The MCDI is a caregiver-report tool used to assess early language skills using two forms: words and sentences (WS) and words and gestures (WG). Participants who were reported to use phrase speech by their caregiver completed the WS form; all others completed the WG form. Because all participants exceeded the normative age ranges for the MCDI (i.e., greater than 30 months for WS; greater than 16 months for WG), a modified scoring approach described in the instrument manual was used to estimate age-equivalent scores up to 30 months. Note that comprehension is not assessed on the WS form.

Intellectual functioning was assessed using the Stanford-Binet Intelligence Scales, Fifth Edition (SB-5 [14]), and adaptive functioning was assessed using the Vineland Adaptive Behavior Scales, Third Edition (VABS-3; [16]). Disease severity was scored using the SLOS severity scale (SSS; [2, 11]). Baseline plasma and CSF levels of total cholesterol, 7-DHC, and 8-DHC were obtained from the simvastatin trial.

All study procedures were approved by the Johns Hopkins Medicine Institutional Review Board (IRB) and conducted in accordance with institutional ethical standards. All assessments were conducted by trained clinicians in a controlled setting. Children from families primarily using languages other than English were excluded, as all instruments were administered in English. Informed consent was obtained from legal guardians of participants, and assent was obtained from participants, when developmentally appropriate.

Descriptive statistics summarize demographic and clinical characteristics. Developmental quotients (DQ) for PPVT and EOWPVT were calculated as follows: DQ = (age-equivalent score/chronological age in months) × 100. Given the small sample size, non-normal distributions, and presence of outliers, nonparametric statistical tests were used. Mann–Whitney *U*-tests were applied to assess group differences by sex, ASD diagnosis, and scorability (scorable vs. non-scorable test data). This includes comparisons of VABS-3 Adaptive Behavior Composite (ABC) scores and Communication Domain (Com) scores across groups and cognitive test completion (SB-5). Spearman’s rank-order correlations were conducted to assess associations between receptive (PPVT) and expressive (EOWPVT) language scores and other variables of interest, including IQ indices, VABS-3 ABC and Com scores, SSS, and biochemical markers (plasma and CSF levels of cholesterol, 7-DHC, and 8-DHC). Fisher’s exact test was used to compare the proportion of ASD diagnoses between participants with scorable versus non-scorable language testing.

Statistical significance was defined as a two-tailed *p*-value < 0.05. To account for multiple comparisons, including 12 planned biomarker-language associations and additional analyses involving intellectual, language, and adaptive functioning measures, a Benjamini-Hochberg (BH) correction was applied to control the false discovery rate (FDR) across 32 total comparisons. FDR-corrected *p*-values are reported where applicable.

## Results

A total of 23 participants were enrolled in the study; however, complete data was available for 21 participants (Table 1). The cohort included 11 males (52.4%) and 10 females (47.6%), with a mean age of 7.85 years (*SD*: 3.0 years). Fifteen participants (71.4%) met criteria for ASD based on DSM-IV diagnostic criteria [1] and the Autism Diagnostic Interview-Revised (ADI-R; [15]), while 6 (28.6%) did not meet diagnostic criteria for ASD. The median SSS was 11, with a range of 6 to 28. No significant differences in SSS were observed between males and females (*U* = 57.5, *p* = 0.88).

**Table 1:** Individual-level demographic, diagnostic, cognitive, and language data for study participants

ID	Sex	Age (y)	ASD	SSS	FSIQ	PPVT SS	EOWPVT SS	ABC	Com
S01	F	10.0	No	11	52	68	69	60	52
S02	F	4.4	Yes	11	52	60	NSB	69	70
S03	M	8.8	Yes	22	40	40	NSB	29	29
S04	M	13.3	Yes	11	48	51	57	45	47
S05	M	8.9	No	11	65	76	73	71	67
S06	M	10.6	Yes	6	56	72	69	53	56
S07	M	7.1	Yes	11	40	NSB	NSB	32	35
S08	M	9.8	Yes	28	INC	NSB	NSB	29	30
S09	F	7.6	Yes	22	47	40	54	50	53
S10	F	12.0	Yes	17	40	40	54	34	26
S11	M	5.6	Yes	6	INC	NSB	NSB	43	44
S12a	F	17.5	Yes	17	INC	40	NSB	19	19
S13	F	10.3	No	6	51	62	76	54	44
S15a	M	4.8	Yes	6	59	80	85	67	71
S16	F	5.4	Yes	22	INC	40	NSB	36	45
S18	M	4.0	No	11	75	90	93	66	86
S19	M	4.1	No	17	94	67	NSB	72	88
S20	M	6.9	Yes	6	INC	NSB	NSB	31	35
S21	F	4.1	Yes	11	58	62	71	53	60
S22	M	13.3	Yes	22	INC	40	NSB	31	19
S23	F	6.1	No	6	83	101	110	78	91

*ABC* (Vineland Adaptive Behavior Scales, 3rd Edition, Adaptive Behavior Composite score), *Com* (Vineland Adaptive Behavior Scales, 3rd Edition, Communication Domain score), *EOWPVT SS* (Expressive One-Word Picture Vocabulary Test, Standard Score), *FSIQ* (Full-Scale IQ, Stanford-Binet, 5th Edition), *INC* (incomplete), *NSB* (non-scorable, basal not obtained), *PPVT* (Peabody Picture Vocabulary Test, Standard Score), *SSS* (SLOS Severity Score).

^a^Active treatment with simvastatin during PPVT/EOWPVT assessment

Two participants (S12 and S15) were receiving active simvastatin treatment in addition to standard-of-care cholesterol supplementation at the time of their language assessments; all other participants were receiving standard-of-care supplementation only.

The SB-5 was successfully administered to 15 participants (71.4%), including 8 males (53.3%) and 7 females (46.7%). The mean nonverbal IQ (NVIQ) score was 58.7 (*SD*: 15.6), mean verbal IQ (VIQ) score was 59.5 (*SD*: 17.1), and mean Full-Scale IQ (FSIQ) score was 57.3 (*SD*: 16.0) (Table 1). No significant differences in NVIQ, VIQ, or FSIQ scores were observed based on sex or SSS. SB-5 testing could not be completed for the remaining six participants (4 males, 2 females) due to profound language impairment, limited cooperation, or global developmental delays that precluded standardized testing. These individuals represent a more severely affected subgroup in which both cognitive and language measures were often non-scorable. The proportion of males and females unable to complete SB-5 testing did not differ significantly (Fisher’s exact test, *p* = 1.00).

All participants completed the VABS-3 (Table 1), with a mean ABC SS of 48.7 (*SD*: 17.5) and mean Com SS of 50.8 (*SD*: 21.9). Lower ABC and Com SS were significantly associated with an inability to complete standardized cognitive and/or language testing. Participants with non-scorable EOWPVT scores (*n* = 10) had significantly lower ABC SS (39.1 vs. 57.4; *U* = 89.0, *p* = 0.018; corrected *p* = 0.035) and trended toward lower Com SS (41.4 vs. 59.4; *U* = 84.5, *p* = 0.04, corrected *p* = 0.063) than those with scorable EOWPVT data. No statistical significance was observed in ABC SS or Com SS for those with scorable compared to non-scorable PPVT. Those who were unable to complete the SB-5 had significantly lower ABC SS (31.5 vs 55.5, *U* = 81.5, *p* = 0.005, corrected *p* = 0.017) and Com SS (32.0 vs 58.33; *U* = 78.0, *p* = 0.011; corrected *p* = 0.026) compared to those who completed the full IQ assessment.

Receptive and expressive language scores were strongly associated with intellectual functioning in this cohort. Among participants with available IQ data, PPVT SS showed robust, statistically significant correlations with NVIQ (Spearman’s rho = 0.76, *p* = 0.002; corrected *p* = 0.011), VIQ (Spearman’s rho = 0.74, *p* = 0.003; corrected *p* = 0.011), and FSIQ (Spearman’s rho = 0.74, *p* = 0.003; corrected *p* = 0.011). Similarly, EOWPVT SS was strongly correlated with NVIQ (Spearman’s rho = 0.79, *p* = 0.002; corrected *p* = 0.011), VIQ (Spearman’s rho = 0.78, *p* = 0.003; corrected *p* = 0.011), and FSIQ (Spearman’s rho = 0.83, *p* < 0.001; corrected *p* = 0.008). In addition, both receptive and expressive language scores were significantly associated with adaptive functioning. Strong positive correlations were observed between ABC SS and PPVT SS (Spearman’s rho = 0.84, *p* < 0.001; corrected *p* = 0.008) and ABC SS and EOWPVT SS (Spearman’s rho = 0.87, *p* < 0.001; corrected *p* = 0.018), as well as between Com SS and PPVT SS (Spearman’s rho = 0.81, *p* < 0.001; corrected *p* = 0.008) and Com SS and EOWPVT SS (Spearman’s rho = 0.76, *p* = 0.007; corrected *p* = 0.019).

Eleven participants completed the MCDI WG Form (mean age: 97.7 months, *SD*: 48.9 months; Table 2). Four participants (S02, S03, S11, S16) had comprehension age-equivalent scores below 16 months; three of whom (S03, S11, S16) also had expressive age-equivalent scores in the same range. One participant (S20) was non-verbal; thus, age-equivalent scores for receptive and expressive language could not be obtained. The remaining six participants achieved the highest age-equivalent bracket of greater than 16 months in both expressive and receptive domains.

**Table 2:** MCDI words and gestures: receptive and expressive age-equivalent scores

ID	Age (months)	Expressive	Comprehension
S02	53	> 16 months	13 months
S03	105	15 months	15 months
S08	117	> 16 months	> 16 months
S09	96	> 16 months	> 16 months
S11	66	11 months	13 months
S12	210	> 16 months	> 16 months
S16	65	< 8 months	8 months
S20	83	NSB	NSB
S21	49	> 16 months	> 16 months
S22	158	> 16 months	> 16 months
S23	73	> 16 months	> 16 months

*NSB* Non-Scorable

Ten participants completed the MCDI WS Form (mean age: 102.9 months, *SD*: 40.9 months; Table 3). Three participants (S15, S18, S19) had expressive language age-equivalent scores between 24 and 26 months, and one participant (S07) scored below 16 months. The remaining six participants reached the test ceiling of greater than 30 months in expressive language.

**Table 3:** MCDI words and sentences: expressive age-equivalent scores

ID	Age (months)	Expressive
S01	121	> 30 months
S04	160	> 30 months
S05	107	> 30 months
S06	127	> 30 months
S07	85	< 16 months
S10	150	> 30 months
S13	124	> 30 months
S15	57	26 months
S18	49	24 months
S19	49	26 months

**Fig. 1:**
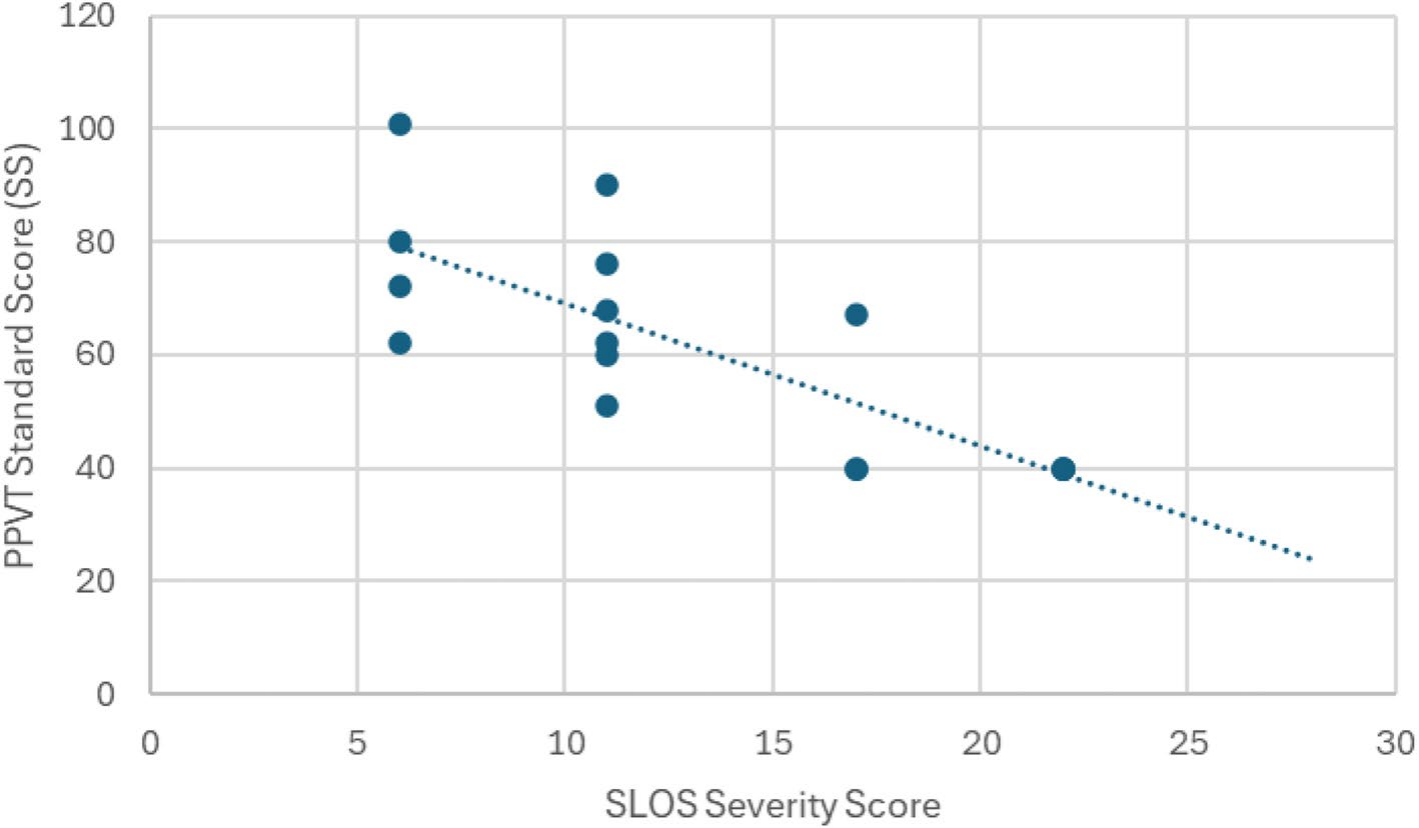
Association between receptive vocabulary scores (PPVT-3) and SLOS severity score

The median PPVT SS among participants with scorable data (*n* = 17) was 62 (range: 40–101), with a median DQ of 41 (range: 8–123). There was no significant difference in median PPVT SS between males and females. A strong, statistically significant negative correlation was observed between PPVT standard scores and SSS (Spearman’s rho = −0.83, *p* < 0.001; corrected *p* = 0.003; Fig. 1).

**Fig. 2:**
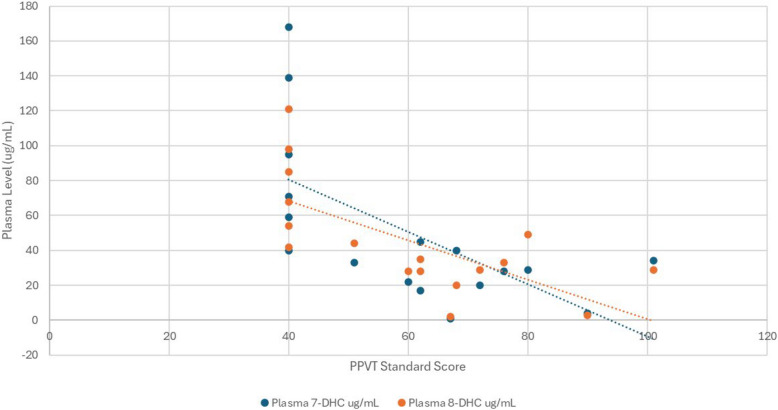
Association between receptive vocabulary scores (PPVT-3) and plasma sterol precursor levels (7-DHC and 8-DHC)

PPVT SS showed a trend towards a significant association with plasma cholesterol level (Spearman’s rho = −0.45, *p* = 0.07; corrected *p* = 0.086) but a nonsignificant association with CSF cholesterol level (Spearman’s rho = −0.32; *p* = 0.21; corrected *p* = 0.23). PPVT SS was significantly negatively associated with plasma sterol precursor levels (7-DHC; Spearman’s rho = −0.56, *p* = 0.017; corrected *p* = 0.035; 8-DHC; Spearman’s rho = −0.55, *p* = 0.018; corrected *p* = 0.035; Fig. 2). Additionally, a significant negative association was also observed between PPVT SS and CSF 7-DHC (Spearman’s rho = −0.55, *p* = 0.02; corrected *p* = 0.035). No significant association was observed between PPVT SS and CSF 8-DHC (Spearman’s rho = −0.35, *p* = 0.17; corrected *p* = 0.22).

**Fig. 3:**
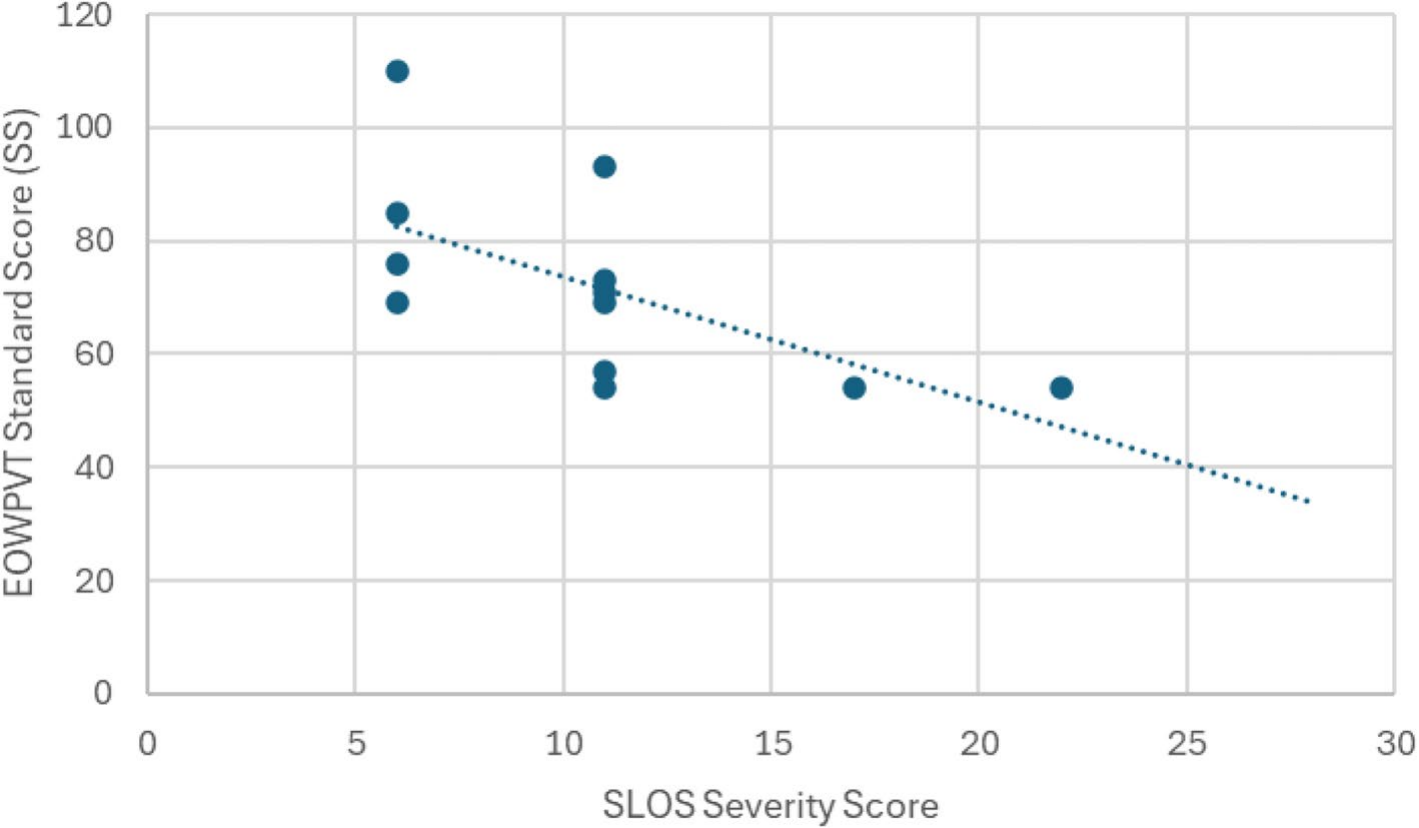
Association between expressive vocabulary scores [3] and SLOS severity score

For participants with a scorable expressive language data (*n* = 11), the median EOWPVT SS was 70 (range: 54–110) with a median DQ of 55 (range: 24–115). No significant difference in EOWPVT SS was observed between males and females. A significant negative correlation was observed between EOWPVT SS and SSS (Spearman’s rho = −0.69, *p* = 0.02; corrected *p* = 0.035; Fig. 3).

No significant associations were observed between EOWPVT SS and cholesterol levels or sterol precursor levels in either plasma or CSF.

Individuals without ASD had significantly higher median PPVT SS compared to those with ASD (72.0 vs. 40.0; *U* = 58.5, *p* = 0.01; corrected *p* = 0.025). There was a trend towards lower EOWPVT SS in individuals with ASD compared to those without (76.0 vs. 63.0; *U* = 25.5, *p* = 0.067; corrected *p* = 0.096). Although all participants with a non-scorable PPVT (*n* = 4) and 90% of those with a non-scorable EOWPVT (*n* = 9) had an ASD diagnosis, the proportion of individuals with ASD did not differ significantly between those with scorable and non-scorable assessments.

A sensitivity analysis excluding participants S12 and S15, who were receiving active simvastatin treatment during language assessment, yielded comparable results. The direction and degree of associations remained stable, indicating that the inclusion of these participants did not meaningfully affect the overall findings.

## Discussion

The results of this study provide important insights into the language development and intellectual functioning of individuals with SLOS, a rare autosomal recessive disorder of cholesterol biosynthesis associated with a broad spectrum of neurodevelopmental impairments.

### Cognitive functioning and sex differences

SB-5 testing revealed low mean IQ scores across the cohort, reflecting the intellectual disability typically associated with SLOS. No significant differences in NVIQ, VIQ, or FSIQ scores were observed between males and females, suggesting that biological sex did not significantly influence cognitive outcomes in this cohort. This finding contrasts with earlier work by Tierney et al. [18], which reported greater cognitive impairment in males with SLOS. One plausible explanation for this discrepancy is diagnostic ascertainment bias. Males are more frequently diagnosed with SLOS due to the presence of distinctive genital anomalies at birth such as micropenis, ambiguous genitalia, or sex reversal, which serve as early clinical clues to the disorder. In contrast, females may go underdiagnosed unless they present with more pronounced systemic or neurodevelopmental manifestations. This bias may result in a female sample that is skewed toward more severe phenotypes, thereby reducing observable sex differences in IQ. Supporting this hypothesis, females in this cohort had severity scores comparable to males, and the overall sex ratio was approximately equal (52.4% male, 47.6% female), unlike the general SLOS population where approximately 70% of diagnosed individuals are male despite the autosomal recessive inheritance pattern [4]. The shift in sex distribution in this cohort may reflect more inclusive recruitment strategies or enhanced recognition of female cases with severe phenotypes. The limited sample size may also have contributed to the absence of observed sex differences by reducing statistical power and potentially obscuring subtle group differences. Together, these considerations suggest that the observed equivalence in cognitive scores across sexes in this cohort may reflect both ascertainment patterns and sampling limitations. Future studies with larger, more representative samples will be critical for clarifying the potential moderating role of sex on neurodevelopmental outcomes in SLOS.

### Receptive language

Approximately, one-fifth of the participants were unable to complete the PPVT due to failure to establish a basal response, rendering these assessments non-scorable. Notably, all individuals with non-scorable PPVT results had a diagnosis of ASD; however, the proportion of participants with ASD did not differ significantly between those with scorable and non-scorable PPVT results, suggesting that ASD alone does not fully account for the observed floor effects. Instead, a strong negative correlation between PPVT scores and SSS supports the interpretation that disease severity may be a major driver of receptive language skills in individuals with SLOS. The SSS reflects the number and severity of congenital malformations observed in SLOS and has been shown to inversely correlate with the ratio of dehydrosterols to total sterols [9, 22], a biochemical marker of disrupted cholesterol biosynthesis. Notably, the SSS includes brain malformation as part of its scoring [11]. In addition to brain abnormalities, individuals with SLOS have been found to have craniofacial anomalies including structural abnormalities of the external and middle ear [10] that may negatively impact hearing and/or auditory processing. In a recent study by Zalewski et al. [23], the most common auditory phenotype was mild to moderate conductive hearing loss, although profound sensorineural hearing loss and retrocochlear dysfunction, evidenced by abnormal auditory brainstem responses, were also observed in approximately 22% of participants. These findings raise the possibility that structural anomalies affecting the brain and auditory system may impair hearing and/or receptive language processing, thereby contributing to floor effects on standardized language assessments. Although formal auditory evaluations were not included in the current study, future investigations should incorporate comprehensive audiological assessments to clarify the role of hearing function in language development and test performance in individuals with SLOS. Integrated neurologic and audiologic assessments may be particularly important for disentangling the contributions of sensory versus cognitive factors on language outcomes in this population.

Notably, over half of the participants with non-scorable PPVT or EOWPVT results also lacked FSIQ scores. All these individuals carried a diagnosis of ASD, but the overlap between missing IQ data and non-scorable language assessments suggests that extremely low cognitive ability may be a common contributing factor. While ASD is associated with language impairment, the inability to establish a basal score on either receptive or expressive vocabulary tasks is more likely reflective of profound intellectual disability or mental ages below the floor of standardized testing. The ADI-R was completed for all participants, including those without formal IQ scores; however, the instrument’s validity may be limited in cases where mental age is below 2 years. This further complicates the interpretation of ASD features in the most severely affected subgroup. Although standardized IQ estimates were not available for these participants with non-scorable language assessments, clinical observations consistently indicated profound global developmental delays in these individuals. This underscores the contribution of cognitive ability to language performance in SLOS and highlights the limitations of conventional standardized assessments in individuals with severe impairments. In support of this, receptive language scores showed strong positive correlations with IQ indices, reinforcing their validity as indicators of general cognitive functioning. Together, these findings emphasize the need for alternative tools that can more accurately assess language and cognitive functioning in profoundly affected individuals who fall below the floor of traditional testing instruments.

### Expressive language

Nearly half of the participants were unable to complete the EOWPVT due to inability to obtain a basal response — reflecting profound deficits in expressive vocabulary. While scorable EOWPVT scores were slightly higher on average than PPVT scores, the greater proportion of non-scorable EOWPVT assessments highlights more frequent and severe impairments in expressive relative to receptive language skills. This pattern is consistent with previous findings in SLOS suggesting that comprehension often exceeds expressive ability [18]. Notably, EOWPVT SS was significantly negatively correlated with SSS, indicating that individuals with more severe clinical features tended to have poorer expressive language performance. This relationship may reflect the broader impact of disease burden, including cognitive and oral-motor impairments, on a child’s ability to verbally produce language, even if comprehension remains relatively more intact.

As with receptive language, the proportion of individuals with ASD did not differ significantly between those with scorable and non-scorable EOWPVT assessments. This suggests that while ASD may contribute to expressive language challenges in individuals with SLOS, it does not fully account for the severity of expressive language impairment observed in this population. The high frequency of non-scorable EOWPVT assessments, including among individuals with ASD, points to broader neurodevelopmental and disease-related factors influencing expressive language outcomes. Furthermore, no associations were observed between expressive language scores and level of plasma or CSF cholesterol, 7-DHC, and 8-DHC. This lack of relationship suggests that variation in expressive vocabulary abilities is not directly explained by current biochemical markers of cholesterol synthesis or sterol precursor accumulation. Instead, expressive language outcomes in SLOS may reflect a more complex interplay of neurodevelopmental burden, cognitive capacity, and motor speech planning deficits that are not fully captured by sterol biomarkers alone. However, it is important to note that these analyses were limited by the relatively small number of participants with scorable EOWPVT assessments. Due to the high rate of floor effects in this cohort, many individuals were unable to achieve a basal score, reducing the sample size available for correlation analysis. This limited subset (*n* = 11) may have reduced statistical power to detect subtle associations between expressive vocabulary and biochemical markers, underscoring the need for cautious interpretation and further investigation in larger, more phenotypically diverse samples.

### Alternative assessment strategies

Given the high proportion of non-scorable language assessments, especially among participants with ASD or severe impairment, the adapted MCDI provided a valuable alternative for estimating language abilities. The MCDI proved feasible to administer across a wide age range and yielded meaningful information on expressive and receptive skills for participants unable to complete standardized testing. This highlights the importance of caregiver-reported tools when assessing development in populations with profound cognitive and communicative delays. Notably, among participants who completed the MCDI WS form, over half reached the test ceiling, suggesting relative expressive strengths in a subset and/or limitations in the tool’s sensitivity at higher ability levels. These findings emphasize the utility of developmentally anchored, caregiver-report measures to complement performance-based assessments, especially in rare genetic syndromes where language profiles are heterogeneous and often fall outside the measurable range of conventional tools.

### Adaptive functioning

All participants completed the VABS-3, and results indicated a significant association between adaptive functioning and the ability to complete standardized cognitive and language assessments. Participants with non-scorable EOWPVT assessments had significantly lower ABC SS compared to those with scorable data. Similarly, individuals who were unable to complete the SB-5 had significantly lower scores on both the ABC and Communication Domain than those who completed the full assessment. These findings suggest that global adaptive functioning, as measured by the ABC, may serve as a general indicator of a participant’s developmental capacity to engage in standardized testing.

Notably, the VABS-3 Communication Domain (Com) appeared particularly sensitive to the feasibility of language-based testing, with lower scores associated with non-scorability on the EOWPVT and inability to complete the SB-5. Furthermore, among participants with scorable test data, EOWPVT and PPVT scores were strongly and positively correlated with the Com SS. This pattern suggests that stronger functional communication skills are meaningfully associated with better performance on both expressive and receptive vocabulary measures. However, the association between Com SS and PPVT scorability did not reach statistical significance, likely due to floor effects and limited score variability among participants with the most severe impairments.

Taken together, these results suggest that while both the ABC and Com SS provide useful information, the Com SS may be particularly valuable for identifying individuals at risk for test non-completion. It not only reflects the functional language abilities necessary for engaging in standardized tasks but also shows strong associations with direct language performance and test feasibility. As such, the VABS-3 Communication Domain may serve as a critical tool in comprehensive evaluation strategies for individuals with SLOS and other populations with profound developmental delay, especially when direct testing is not feasible.

### Cholesterol metabolism and broader neurodevelopmental implications

The findings from this study, together with those from Thurm et al. [17], contribute to a more nuanced understanding of the relationship between cholesterol metabolism and language functioning in SLOS. In our sample, receptive language performance as measured by the PPVT was only weakly and nonsignificantly associated with plasma or CSF cholesterol levels, consistent with prior findings from Thurm et al. [17], which included all subjects from this study. Thurm and colleagues reported that plasma cholesterol showed stronger correlations with cognitive and adaptive functioning than CSF cholesterol, suggesting that systemic cholesterol may be more relevant to global neurodevelopmental outcomes.

In contrast to total cholesterol, we observed significant negative associations between PPVT SS and plasma sterol precursors — specifically 7-DHC and 8-DHC — as well as with CSF 7-DHC. These findings indicate that a higher sterol precursor burden is associated with poorer receptive language performance. Notably, this pattern was not observed for CSF 8-DHC, which showed a nonsignificant association with PPVT SS. Taken together, these results suggest that precursor sterol accumulation, which reflects the underlying enzymatic disruption in the cholesterol biosynthetic pathway, may serve as a more sensitive biomarker of receptive language impairment in SLOS than total cholesterol levels. The absence of significant associations with CSF cholesterol, as also reported by Thurm et al., may reflect limitations related to measurement variability, smaller sample size, or a weaker mechanistic link to language outcomes compared to sterol precursors.

Expressive language scores, measured by the EOWPVT, did not show significant associations with biochemical markers of cholesterol metabolism in this study. Although the direction of association was negative for several sterol markers, these findings did not reach statistical significance, suggesting a weaker or less consistent relationship between expressive vocabulary and cholesterol pathway disruption compared to receptive language. Nevertheless, the overall pattern observed across language domains — particularly the significant associations between PPVT scores and sterol precursors — reinforces the potential role of impaired cholesterol biosynthesis in shaping language outcomes in SLOS. Further studies in larger cohorts are needed to clarify these relationships and identify which biochemical markers most closely track with specific language impairments.

Importantly, the current study included a substantial overlap with the Thurm et al. [17] dataset, with 20 of 26 CSF samples and 21 of 30 serum samples derived from individuals in our cohort. This overlap strengthens confidence in the replicability and robustness of the observed biomarker-language associations, particularly those involving sterol precursors. The consistent findings across studies highlight the value of serum-based sterol measures as more accessible and informative indicators of neurodevelopmental status. In contrast, CSF cholesterol, while theoretically more proximal to brain biochemistry, was more invasive to obtain and showed weaker or inconsistent associations with developmental outcomes in both datasets.

Beyond the context of SLOS, our findings contribute to a growing body of evidence linking cholesterol metabolism to neurodevelopmental outcomes more broadly, particularly in ASD. Within our cohort, individuals without ASD had higher receptive vocabulary scores than those with ASD. This trend aligns with the language and cognitive vulnerabilities commonly associated with autism, though findings should be interpreted cautiously given the small sample size and lack of significance after multiple comparison adjustment. Importantly, an ASD diagnosis did not fully explain the floor effects observed with standardized testing, indicating that other biological factors, such as disease severity or cholesterol metabolism, may also contribute. This link is further supported by prior work demonstrating abnormal lipid profiles in subsets of individuals with ASD. Tierney et al. [19] reported significantly low serum cholesterol levels in children with ASD, while Luo et al. [13] identified a lipid metabolism-associated subtype of autism based on alterations in lipid profiles in affected children and their parents. More recently, Esposito et al. [6] conducted a comprehensive review of 36 studies and confirmed a significant correlation between ASD and hypocholesterolemia, suggesting that impaired cholesterol homeostasis may be a feature of a biologically distinct ASD subgroup. Tierney et al. [20] further extended this work, finding that HDL cholesterol and apolipoprotein A1 were positively correlated with adaptive functioning in individuals with ASD from multiplex families. That study also identified a subgroup of ASD individuals with biochemical features suggestive of clinical lipid disorders, including markedly reduced levels of 7-DHC, 8-DHC, lathosterol, desmosterol, and sitosterol — findings consistent with impaired cholesterol synthesis. Taken together, these findings suggest that cholesterol and its precursors may serve not only as biomarkers for developmental outcomes but also as targets for intervention, including dietary or pharmacologic strategies aimed at restoring lipid balance.

Our findings from the SLOS population, where cholesterol metabolism is severely impaired, add weight to this hypothesis and suggest that metabolic profiles may be central to understanding language impairments and broader neurodevelopmental trajectories in both rare and idiopathic conditions. As such, integrating metabolic biomarkers into clinical assessment protocols may offer a pathway toward more personalized approaches to evaluation and treatment in neurodevelopmental disorders.

### Limitations

While this study provides important insights into the neurodevelopmental profile of individuals with SLOS, several limitations must be acknowledged. First, the relatively small sample size, though expected in rare neurodevelopmental disorders, limits statistical power and may reduce the generalizability of findings. In addition, a substantial proportion of the participants overlapped with prior studies, such as Thurm et al. [17], which could introduce potential bias. This overlap may lead to an overestimation of effect sizes, distortion of correlation estimates, or loss of statistical independence. Selection bias may also be a concern, particularly if individuals included in multiple studies differ systematically from those not captured in either sample.

The cross-sectional nature of the data further constrains interpretation, as it reflects only a single time point and precludes conclusions about developmental trajectories or causal relationships. Group-level analyses, while necessary, also risk obscuring important individual variability, an especially relevant concern in syndromic populations like SLOS, which are marked by significant phenotypic heterogeneity. Additionally, because many participants could not complete standardized cognitive or language assessments, alternate measures such as caregiver-report tools were used. While these are clinically valuable, they may not fully substitute for direct assessment and limit comparability across tools. Finally, other potential confounding variables, such as genetic modifiers, environmental exposures, or medication effects, were not analyzed and may influence outcomes.

### Future directions

Future research would benefit from larger, longitudinal cohorts capable of capturing developmental changes over time and characterizing intraindividual variability more precisely. Such designs could help disentangle the individual and combined effects of disease severity, cholesterol metabolism, and co-occurring ASD on cognitive and language outcomes in SLOS. Incorporating naturalistic or ecologically valid methods, such as language sampling, may also provide a more comprehensive understanding of functional communication in this population.

In conclusion, our findings indicate that speech and language performance in SLOS is associated with multiple factors, including disease severity, disrupted cholesterol metabolism, and co-occurring ASD. Although these factors were examined independently in the current study, future research should explore potential interactions to better understand their collective influence on developmental outcomes. Collectively, these results contribute to our understanding of the biological underpinnings of neurodevelopment in SLOS and may inform future research and clinical strategies aimed at improving cognitive and language outcomes in individuals with neurodevelopmental disorders.

## Data Availability

No datasets were generated or analysed during the current study.
